# Current Status of Patient-Derived Ovarian Cancer Models

**DOI:** 10.3390/cells8050505

**Published:** 2019-05-25

**Authors:** Yoshiaki Maru, Yoshitaka Hippo

**Affiliations:** Department of Molecular Carcinogenesis, Chiba Cancer Center Research Institute, Chiba 260-8717, Japan; ymaru@chiba-cc.jp

**Keywords:** ovarian cancer, patient-derived cells, organoid, spheroid, xenograft, pre-clinical model, precision medicine, drug discovery

## Abstract

Ovarian cancer (OC) is one of the leading causes of female cancer death. Recent studies have documented its extensive variations as a disease entity, in terms of cell or tissue of origin, pre-cancerous lesions, common mutations, and therapeutic responses, leading to the notion that OC is a generic term referring to a whole range of different cancer subtypes. Despite such heterogeneity, OC treatment is stereotypic; aggressive surgery followed by conventional chemotherapy could result in chemo-resistant diseases. Whereas molecular-targeted therapies will become shortly available for a subset of OC, there still remain many patients without effective drugs, requiring development of groundbreaking therapeutic agents. In preclinical studies for drug discovery, cancer cell lines used to be the gold standard, but now this has declined due to frequent failure in predicting therapeutic responses in patients. In this regard, patient-derived cells and tumors are gaining more attention in precise and physiological modeling of in situ tumors, which could also pave the way to implementation of precision medicine. In this article, we comprehensively overviewed the current status of various platforms for patient-derived OC models. We highly appreciate the potentials of organoid culture in achieving high success rate and retaining tumor heterogeneity.

## 1. Introduction

Ovarian cancer (OC) is the most devastating gynecologic cancer. Even after development of many surgical techniques and chemotherapies, its overall five-year survival rate is still as low as 47% [1]. The prognosis appears favorable if patients are diagnosed at early stages, but early detection is generally difficult due to manifestation of non-specific symptoms and the lack of reliable biomarkers. 

With many histological variations, OC has been regarded as a highly heterogeneous disease. Indeed, epithelial OC that comprises nearly 90% of OC is usually classified into many histologically distinct subtypes. Major subtypes include serous carcinoma, endometrioid carcinoma, clear cell carcinoma, and mucinous carcinoma. Recently, epithelial OC are more loosely classified into two general categories by integrating the results from histopathological, molecular biological, and genetic analyses, to underscore biological properties of OC. Type 1 cancers grow slowly and contain low-grade serous carcinoma (LGSC), mucinous carcinoma, endometrioid carcinoma, clear cell carcinoma, and transitional carcinoma. Type 2 cancers progress rapidly and contain high-grade serous carcinoma (HGSC), undifferentiated carcinoma, and carcinosarcioma [2]. This dualistic model of ovarian cancers was further revised and expanded to provide more detailed classification [3]. Researchers used to automatically assume that most epithelial OC would derive from ovarian surface epithelium (OSE), but accumulating evidence strongly suggests that its cell of origin might differ by histological subtypes. For example, it has been established that HGSC predominantly originates from secretory cells or progenitor cells in the fallopian tubes, via pre-malignant lesions known as serous tubal intraepithelial carcinoma (STIC) [4]. Endometrioid carcinoma and clear cell carcinoma are highly correlated with a history of endometriosis, in which endometrial cells implanted on the ovary are the likely origin of the tumors [5,6]. Genome analysis revealed that differences in mutation profiles underlie histological subtypes. Whereas *TP53* is exclusively mutated in HGSC in a proportion as high as 95% [7], *RAS* and *BRAF* are frequently mutated in LGSC [8]. *KRAS* mutation is also implicated in mucinous carcinoma [9]. Clear cell carcinoma is characterized by *ARID1A*, *PIK3CA*, *TERT* promoter mutations [10,11,12], and endometrioid carcinoma is characterized by *PTEN*, *PIK3CA*, *ARID1A*, and *CTNNB1* mutations [13]. However, some tumors are not consistent with these typical features, hence molecular mechanisms underlying carcinogenesis of each OC subtype are not fully understood.

Despite the highly heterogeneous nature of OC, standard treatment of ovarian cancer is stereotypically composed of aggressive surgery followed by platinum-taxane chemotherapy. Of the four major subtypes, clear cell carcinoma and mucinous carcinoma tends to be refractory to chemotherapy [14,15]. Moreover, recurrence after initial chemotherapy often results in platinum-resistant diseases, leading to low overall five-year survival rates. To overcome this issue, some new therapeutic agents are in trial for OC. Representative examples include PARP inhibitors for cases deficient in homologous recombination repair, often caused by inactivation of *BRACA1* or *BRCA2* [16], and molecular targeted agents against vascular endothelial growth factor (VEGF) [17]. Nonetheless, treatment options of ovarian cancer are still limited, requiring new therapeutic options. For efficient drug discovery, preclinical models that accurately mimic biological properties of in vivo human tumors would be of great value. In this regard, patient-derived materials are currently becoming indispensable and will also be useful in precision medicine. Along with recent implementation of precision medicine, high-throughput genome sequencing analysis has been applied to explore effective therapeutic strategies for each patient [18]. However, identification of druggable targets may not necessarily warrant efficacy of the drug in a clinical setting. Assays with patient-derived cells, by direct administration of drugs to cells in vitro or to xenografts, would therefore be helpful in predicting drug response. 

**Figure 1:**
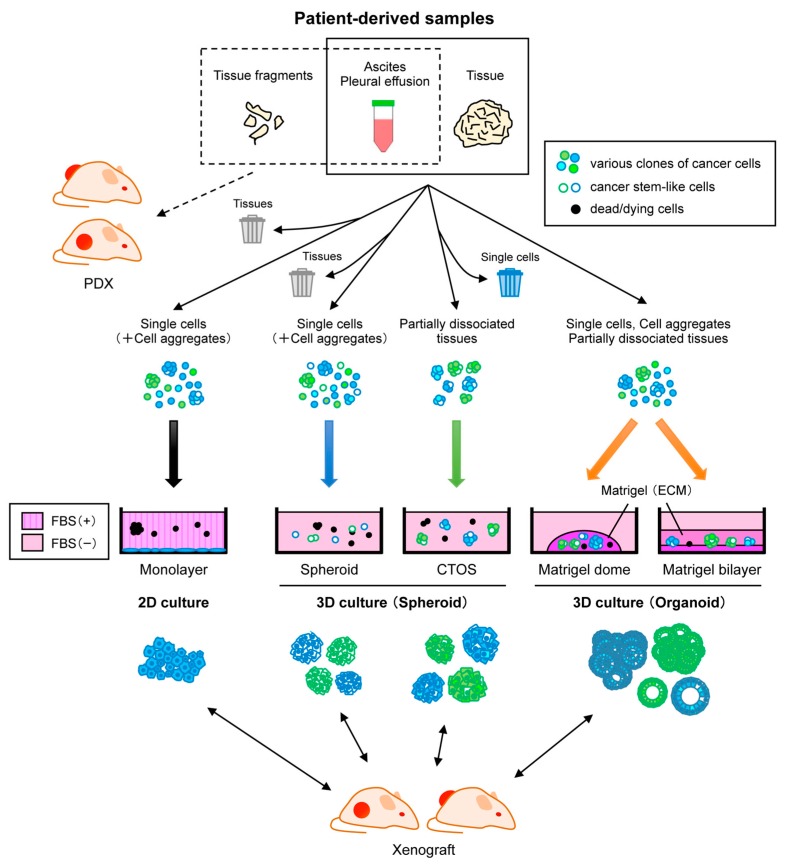
Representative approaches for establishing patient-derived cancer models from diverse clinical samples. Patient-derived xenografts (PDXs) are generated by direct engraftment of clinical samples into immunodeficient mice. Monolayer culture is a common culture method, but cells from primary tumors often undergo crisis, leading to positive selection of specific clones. Spheroid culture with serum-free media is suitable for enrichment of cancer stem-like cells. Cancer tissue-originated spheroids (CTOS) method initiates culture by maintaining cell-cell contact of cancer cells. In the presence of extracellular matrix (ECM) such as Matrigel, organoid culture can propagate both normal and cancer cells while retaining heterogeneity and differentiation. CTOS of ovarian cancer have been not documented yet. These cells cultured by various methods can be used to generate xenografts.

Such patient-derived models, especially primary cell culture, have not been intensively developed for OC thus far, unlike for cancers of other vital organs. It is not clear whether this is because of any technical difficulties specific to OC or researchers simply did not attempt to obtain patient-derived material for OC. In this article, we comprehensively overview the current status of various patient-derived platforms (Figure 1) and illustrate pros and cons of each system in OC to gain perspectives on potential issues to be circumvented in OC research. 

## 2. Cancer Cell Lines

### 2.1. General Overview

Cancer cell lines are special types of cells that acquire infinite proliferation potential on plastic dishes. They can expand as a monolayer sheet, with simple media that typically contains fetal bovine serum (FBS). For their high usability as adherent cells, cancer cell lines have long contributed to many areas of scientific research, including cell biology, biochemistry, physiology, and drug discovery, let alone cancer research [19]. To better interpret the results of experiments with cell lines, the Cancer Cell Line Encyclopedia (CCLE) has been released [20]. This catalog accommodates various molecular profiling, including genome and transcriptome, for around 1000 widely used cell lines derived from various cancer types. This resource will be helpful in linking experimental observations and clinical significance.

On the other hand, there are several drawbacks in cell lines. Firstly, they tend to be passaged too many times. As the success rate for establishing cell lines from primary tumor tissues is generally low, only a subset of cell lines has been extensively used, thereby cultured over a significantly long period of time or many passages. With the high potential of FBS-based cultures to introduce mutations and genome instability, cell lines could undergo drastic changes in terms of morphology and biological properties, presumably by selection of some specific clones. Consequently, even cell lines under the same name could harbor significant diversity between laboratories [21]. Diversity could arise even within the same laboratory between early and late passage. For example, as xenografts in immunodeficient mice, adenocarcinoma-derived cell lines may no longer exhibit a glandular structure and even exhibit undifferentiated histology. Secondly, there is a cross contamination issue in commonly used cell lines. As is so often observed, even cell lines from a large public-sector cell bank could suffer from this issue. Consequently, requirements for authentication of cell lines have become increasingly strict [22]. Presumably due to these two reasons, the responses of some cell lines to therapeutic agents, either in monolayer culture or as xenograft, were not recapitulated in many clinical trials [23,24], discouraging the use of cell lines as a pre-clinical model. 

### 2.2. Ovarian Cancer Cell Lines

More than 50 OC cell lines have been so far established and are currently available for research. As predicted, discrepancies in histology or mutation profiles between cell lines and original tumors were documented in a study that investigated in detail the validity of 47 OC cell lines derived from various OC subtypes [25]. Among the Pubmed literature that used any of these cell lines, the studies using SK-OV-3, A2780, OVCAR-3, CAOV3, or IGROV1 as HGSC models held a share of 90%. However, genomic profiling of SK-OV-3 and A2780 analysis demonstrated *TP53* was intact, although this is a hallmark mutation in HGSC. On the other hand, it detected mutations in *ARID1A*, *BRAF*, *PIK3CA*, and *PTEN*, which are commonly mutated in other subtypes of OC. Besides, OVCAR-3 and CAOV3 were not among the top-ranking HGSC cell lines based on integrative molecular profiling, although they in fact possessed *TP53* mutations and substantial copy number change. IGROV1 had a hypermutated phenotype, which is frequently observed in endometrioid carcinoma rather than in HGSC. Strikingly, the 12 cell lines evaluated as best candidates for HGSC models accounted for only 1% of the Pubmed literature on the 47 analyzed cell lines. Morphological characterization of 39 OC cell lines also led to a similar conclusion that questioned SK-OV-3, S2780, and IGROV1 as representative models for HGSC [26]. These results strongly suggest that the most widely used "HGSC" cell lines might not in fact represent HGSC. In some non-serous OC cell lines, considerable discrepancies in gene mutations from earlier studies were observed as well [25], suggesting that the diversitification of the cell lines is another critical issue in OC research. 

Tumorigenicity assay was conducted by inoculating 17 OC cell lines into the subcutis, peritoneal cavity, and ovaries of nude mice, revealing that the tumorigenicity varied among inoculation sites and cell lines [27]. For example, whereas OVCAR-3 did not proliferate in the subcutis, but formed tumors in the peritoneal cavity, HeyA8 formed tumors in either location. The integrated proteomic analysis has divided 26 OC cell lines into three categories: epithelial type, clear cell type, and mesenchymal type, and identified a protein signature that could potentially uncover the cell of origin for each OC subtype and corresponding driver proteins [28]. Collectively, it is therefore advisable to select OC cell lines appropriate for the purpose of the study by carefully examining the characteristics of the cell lines.

## 3. Patient-Derived Xenografts (PDXs)

### 3.1. General Overview

Patient-derived xenografts (PDXs) are usually generated by directly engrafting tumor fragments into immunodeficient mice. Not only surgically resected tumors, but also samples from biopsy, ascites, and pleural effusion can be used. Inoculation sites are most commonly subcutaneous tissues. Depending on the tumor types and purposes of the experiments, orthotopic engraftment or intraperitoneal injection could be an option. Established PDXs can be serially engrafted to multiple mice without a culture step in between. Starting from primary tumors, the tumor take rate of PDX is generally higher than the success rate of establishing cancer cell lines. One possible explanation is that microenvironments reconstituted by interactions between subcutaneous tissues and tumor fragments might be physiological, accelerating tumor development faster than in cell lines, with only epithelial cells in an artificial condition. PDXs have been established from various types of cancer [29]. In most cases, histological features of the original tumors were basically retained, in terms of tissue structure and microscopic findings, including gland structures, mucin production, and cystic development. Moreover, PDXs also retained genetic aberrations and gene expression profiles of the original tumors [30], even after serial in vivo passages [31]. Importantly, reflecting these features, high correlation between therapeutic efficacy in PDX model and patients was documented [32]. These findings established PDXs as an indispensable preclinical model in drug discovery. However, the take rate significantly differs among cancer types and it is still challenging to efficiently develop PDXs from certain cancers, including breast [33] and prostate cancer [34]. Accordingly, optimization of the protocol for each tumor type seems necessary.

On the other hand, there are downsides to PDXs. Firstly, maintaining tumors in immunocompromised mice might be costly and require a more specialized skill in serial passage in mice, compared to simple monolayer cultures of cell lines. Besides, mice with more severely compromised immunity than nude mice might be eventually required to increase the tumor take rate. These mice include non-obese diabetic/severe combined immunodeficiency (NOD/SCID) mice, and NOD/SCID/IL2Rγ^null^ (NSG) mice, which could further involve a significant cost. For that reason, use of PDXs for high-throughput drug screening might be limited. Secondly, PDXs often require long latency to be established [35], and could therefore undergo tumor clonal evolution in a way to adapt to microenvironments in mice [36]. In principle, this effect may be inevitable to this experimental system, and needs to always be taken into account in interpretation of the results of any assays. But researchers have identified such mouse-specific signatures, potentially paving the way to subtract these artificial effects from the data. Thirdly, interactions between PDX and immune systems are totally different from those in humans. As mice lacking functional elements of immune systems are used for generation of PDX, critical differences lie in both species and immunity. To circumvent these issues, humanized mice have been developed. In these immune-deficient mice, CD34^+^ cells isolated from blood of the same patient were intravenously inoculated to reconstitute a functional human immune system [37]. PDXs with patient-matched immune systems may be valuable models for the evaluation of immune checkpoint inhibitors. 

### 3.2. PDX for Ovarian Cancer

The first xenograft model for OC was described back in 1977, in which tumor tissue was engrafted into subcutaneous tissue of nude mice [38]. Several years later, PDXs were also established by intraperitoneal inoculation of tumor tissue or ascites-derived cancer cells [39], and orthotropic engraftment into the capsule of an ovary [40]. Since then, many studies have documented OC PDXs. As a large-scale study of OC PDXs, more than 150 OC PDXs have been established by intraperitoneal inoculation into SCID mice with a take rate of 74% [41]. Implantation sites have been shown to affect the take rate of clinical OC samples [42], being relatively high in non-orthotropic sites such as subcutis and renal capsule, although these sites lack the microenvironment inherent to ovary or peritoneum [42,43]. Orthotropic OC models have several advantages over the models in other sites, because it can recapitulate tumor growth, metastasis, and ascites formation as observed in human disease [44,45,46]. OC cells in ascites were transduced ex vivo with luciferase and intraperitoneally inoculated to generate 14 OC PDX models [46], enabling non-invasive and simple evaluation of tumor burden. PDXs basically retained histological features and genome aberrations of the original OC. Genetic stability of OC xenografts was demonstrated even after serial transplantation into the renal capsule of NOD/SCID mice [47]. In mRNA profiling, HGSC PDXs successfully recapitulated three out of four human HGSC subtypes that were previously proposed by TCGA [7], with an exception for the immunoreactive subtype [48], which obviously requires the presence of intact immune system. As mRNA in the PDX could be derived from both murine and human cells, an efficient bioinformatics method was developed to extract only human transcripts from PDX transcriptome data obtained by RNA sequencing. It revealed that differentially expressed genes between the human tumors in PDX and the original tumors were mostly implicated in stromal components, suggesting the lack of human stroma might both directly and indirectly affect transcriptome of human components in the PDX [49]. 

The responses to platinum chemotherapy in OC PDXs were highly correlated with those in the donor patients [41,50,51,52,53], underscoring the validity of PDX as a preclinical model. Naturally, several potential candidates for therapeutics have been tested on PDXs established from platinum-resistant OC in an effort to overcome drug-resistance [51,53,54]. OC PDXs were also used to test the efficacy of targeted therapy [55]. For example, sensitivity to inhibitors targeting PARP, CHEK1, or ATR was investigated in BRCA-mutated OC PDXs to optimize therapeutic strategies for homologous recombination-deficient OC [56,57]. Hedgehog pathway, upregulated in a subset of OC [58], was also targeted with a specific inhibitor, leading to significant decrease of tumor volume in OC PDX [59]. More recently, combinatorial therapy including both chemotherapeutic agents and HER2 inhibitors was evaluated on OC PDX [60]. Furthermore, tumorigenicity as xenografts per se proved to be inversely correlated with progression-free survival [61]. Based on these findings, the potential relevance of PDX in OC is highly appreciated in terms of both drug discovery and precision medicine. One caveat with OC PDXs is that they predominantly originate from HGSCs, and take rate and latency for other histological subtypes is low and long, respectively [41,53,62]. These issues are to be addressed for the future improvement of this approach to OC in general.

## 4. Patient-Derived Cells

### 4.1. Two-Dimensional Adherent Culture

#### 4.1.1. General Overview

As mentioned earlier, establishment of cell lines from primary cultures of patient-derived samples has only a low efficiency. For example, only 10 breast cancer cell lines were established out of 135 resected primary tumors [63]. Other researchers also established 18 breast cancer cell lines, but with a success rate of 10% [64]. This inefficiency might be partly due to the challenging adaptation of primary cancer cells to an adhesive monolayer culture. Cancer cells frequently lose growth potential after some passages and go into crisis, suggesting that overcoming replicative senescence might be a critical step in becoming a cell line under culture conditions with FBS. Even in successful cases, loss of tumor heterogeneity and proliferation of specific clones were commonly observed during in vitro adaptation. In a long-term serial passage of uveal melanoma (UM) cell lines, severe reduction of the expression of genes encoding UM markers MART-1, and p16 was observed [65]. 

#### 4.1.2. Ovarian Cancer Cell Lines

Intriguingly, 25 novel cell lines were recently established from primary OC with significantly high efficiency, by using culture media and conditions optimized to each histological subtype [66]. More importantly, it was shown that these established cells retained the genomic landscape, histopathology, and molecular features of the original tumors. While how such a high success rate could be achieved needs to be clarified, if this method proves robust enough, it will benefit a broad area of cancer research.

### 4.2. Three-Dimensional Culture

#### 4.2.1. Spheroid Culture

##### General Overview

Spheroids are sphere-like cell aggregates, which are usually maintained as floating three-dimensional structures. Among various spheroid culture methods so far documented [67], the standard protocol is as follows. Tumor tissues are subjected to physical and enzymatic dissociation, followed by filtration with cell strainers, or cell sorting by flow cytometry with stem cell markers. Subsequently, obtained single cell suspensions are cultured in low-attachment plates with serum-free media. Tumor-derived spheroids can develop from a single cell or cell aggregates. Culture supplements include growth factors, such as epidermal growth factor (EGF) and fibroblast growth factor (FGF). It is generally accepted that cell populations with stem cell-like properties will be functionally enriched by spheroid culture. As cancer stem cell theory argues that malignant phenotypes of cancer are mainly mediated by such stem cell-like fractions, spheroid cultures are most commonly adopted in investigations of drug resistance and metastasis [68,69,70]. Spheroids can be also used to generate PDXs, thereby evaluating the tumorigenicity of the original tumors. However, normal epithelial cells do not grow in spheroid culture conditions, therefore there is a lack of a reference sample on the same platform.

##### Ovarian Cancer Spheroids

For OC patients, drug resistance and peritoneal dissemination accompanied by cancerous ascites are two major factors that could profoundly affect prognosis. Given the morphological similarity between tumor cell aggregates within cancerous ascites and tumor spheroids in culture, it was natural that researchers initiated spheroid culture studies of OC with cancerous ascites samples. As was observed in other types of cancer, only a subset of ascites-derived cancer cells exhibited stem-cell like properties, which indeed developed tumors in immune-deficient mice [71]. Resected tumor samples from five ovarian HGSC were subjected to spheroid culture, and cell surface protein CD117 and CD44 were identified as tumor-initiating cell markers [72]. Detailed analysis of spheroids revealed a reciprocal regulatory circuit involving ALDH1 and SOX2 in OC stem cells [73]. Drug sensitivity for cisplatin, ALDH inhibitors, and the JAK1/2 inhibitor ruxolitinib was also evaluated using a hanging drop spheroid culture of ALDH1^+^ CD133^+^ stem cells derived from cancerous ascites of OC patients [74]. Throughout these studies, serous carcinomas of advanced stage, predominantly obtained from ascites, were exclusively used as sources of spheroids. Future refinement of protocols will therefore be necessary to extend the application of this approach to other types of OC.

##### Non-Single Cell-Based Approaches

For spheroid culture, standard preparation of tumor samples begins by obtaining singly dissociated cells. However, this was originally optimized for hematopoietic cells or neuronal cells, by which stem cell biology has considerably evolved. In sharp contrast, destruction of cell-cell adhesion could readily induce anoikis for epithelial cells, which significantly lowers the success rate of spheroid formation for many types of tumors of epithelial origin. To address this issue, only partially digested colorectal cancer (CRC) tissues were selectively subjected to spheroid formation, resulting in substantial improvement of the success rate of primary culture [75]. This approach, designated as the cancer tissue-originated spheroids (CTOS) method, has been successfully applied to various tumor tissues, including cancer of the colon [75], endometrial [76], and lung [77]. Established CTOS basically retained features of the original tumors and was feasible for in vitro high-throughput drug screening with CRC samples [78]. As CTOS do not grow beyond a certain volume, they are usually required to be physically fragmented, but not completely dissociated into single cells, for efficient passage. Alternatively, CTOS can be diverted to xenografts for extensive propagation of cancer cells, which can be in turn switched to CTOS again. While OC-derived CTOS has not been documented yet, given the simple and robust nature of the method, its application to patient-derived OC samples will be expected in the near future. With another culture technique, in which minced tumor tissues were used for spheroid culture for three to six months, 12 spheroid cell lines were established, and high-throughput assay for anti-cancer agents conducted [79].

#### 4.2.2. Organoid Culture

##### General Overview

An organoid is an emerging concept that is literally a mini-organ exclusively composed of epithelial cells. Without the aid of a stromal niche, even a single normal stem cell can differentiate into all the lineages of the organ, while stem cells self-renew, thereby reconstituting in vivo homeostasis of the whole organ. In 2009, it was first demonstrated that murine intestinal stem cells marked by LGR5^+^ could infinitely proliferate in vitro [80]. This study was based on the idea that stem cell niches would be alternatively reconstituted without stroma by defined factors and matrices. Intestinal stem cells are characterized by an activated Wnt pathway and inhibition of the bone morphogenetic protein (BMP) pathway. Besides, it was known that extracellular matrix laminin is enriched in the niche. Specifically, isolated intestinal crypts were embedded in Matrigel with abundant laminin, and cultured in a serum-free media supplemented with R-spondin 1, which is a WNT agonist and ligand of LGR5, epidermal growth factor (EGF), and the bone morphogenetic protein (BMP) inhibitor Noggin. Consequently, tissue homeostasis was reconstituted in vitro and the stem cells formed self-organizing organotypic structures referred to as organoids. Under the same concept, the original experimental protocol was further optimized for various murine and human normal organs [81,82,83]. Its application has been extended to many fields, including bacterial infection [84], developmental biology [85], and epithelial regeneration [86]. 

##### Organoid-Based Carcinogenesis Model

Organoids can be subjected to genetic engineering by viral introduction of shRNA and cDNA. With highly efficient lentiviral gene transduction into murine organoids [87], we demonstrated that the whole processes of multi-step carcinogenesis could be recapitulated for the intestine, lungs and the biliary tract, as subcutaneous tumors in nude mice [88,89,90]. Essentially similar results to earlier in vivo studies were obtained in a significantly shorter period of time, suggesting that this approach might at least partly substitute and complement the conventional gene-targeting approach in modeling carcinogenesis. Similarly, multiple genetic alterations were reconstituted in human colon organoids with CRISPR/Cas9 technology to generate full-blown tumors, although premalignant or benign lesions were not recapitulated in immunodeficient mice [91,92]. These organoid-based carcinogenesis models might be useful for elucidation of the molecular mechanisms underlying carcinogenesis. 

##### Primary Tumor Organoid

Organoid culture techniques can be applied to various patient-derived samples, such as resected tissue, biopsy, ascites, and pleural effusions. Obvious advantages of organoid culture over PDX and spheroid culture include that it allows propagation of normal cells and precancerous cells, and a higher success rate for cancer cells. In CRC, the success rate of organoid culture was significantly higher than that of PDX or spheroid culture [93]. Given that cancer is not an organ in a strict sense, one might argue that it may not be adequate to use the term "organoids" for cancer cells. However, many researchers stretched the interpretation of the term by taking cancer stem cells as an analogy of tissue stem cells. Accordingly, patient-derived cells propagated in Matrigel were defined as tumor-derived organoids in this review. On the other hand, some researchers appear to go beyond that definition, by referring to patient-derived cells that were cultured by any 3D culture methods, spheroids for instance, as “organoids”. Such confusion in nomenclature needs to be taken into account when looking into the literature.

Establishment of tumor-derived organoids from various cancer types has been reported, namely colon [94], pancreatic [95], gastric [96], prostate [97], breast [98], esophageal [99], bladder [100], and endometrial cancers [101]. Whereas the success rate of organoid culture varies by cancer types and tumor grade, it reaches 80 to 90% in colon cancer. Propagated tumor-derived organoids basically retain both histological and genetic features of original tumors and are feasible for in vitro drug sensitivity assay [102], which recapitulates clinical responses of matched patients [103]. Collectively, tumor-derived organoids will likely serve as a promising resource for evaluating clinical response of individual patients. In addition, tumor-derived organoids were used in high-throughput drug screening for precision medicine. For example, drug screening of a library containing more than 50 compounds was conducted with 19 colorectal cancer organoids, demonstrating correlation between drug sensitivity and genetic aberrations [104]. Thirty-seven anti-cancer drugs were screened in nine gastric cancer organoids, which identified a good response toward some new target drugs [96]. Also, various applications are under way, such as secondary establishment of xenografts and single cell analysis [105]. 

On the other hand, there are several shortcomings in organoid culture. Firstly, tumor-derived organoids lack stroma, immune cells, and blood vessels. To address this issue, some co-culture systems involving pancreatic ductal adenocarcinoma-derived organoids and murine pancreatic stellate cells was established to investigate interactions between cancer cells and cancer-associated fibroblasts [106]. Recently, tumor sensitivity to T cell-mediated immune response was also evaluated, by co-culturing colorectal cancer or non-small cell lung cancer-derived organoids and peripheral blood lymphocytes [107]. Secondly, organoid culture is costly in comparison with conventional 2D culture due to supplemental factors and extra cellular matrix such as Matrigel. However, conditioned medium derived from L-WRN cells, which secrete Wnt3a, R-spondin3, and Noggin, became available for organoid culture, at least partially circumventing this issue [108,109]. Thirdly, the success rate of organoid culture is not high enough in a subset of tumor types. Besides, establishment of organoids from tiny clinical samples is still technically challenging. However, tumor-derived organoids could be established from biopsy samples [110], suggesting improvements in success rate. Similarly, further improvement of organoid culture technique might ultimately enable establishment of organoids from circulating tumor cells and tumors spread in spinal fluid. Lastly, culture and assay protocols currently vary among researchers and laboratories, even for the same cancer types. Such differences might potentially affect the outcomes of drug screening assays.

##### Ovarian Cancer Organoid

Unlike many other types of cancer, only a few studies have documented organoid cultures of OC (Table 1). Drug response of OC cells in monolayer cultures and organoid cultures was compared by using cancer cells collected from tumor tissues, ascites and pleural effusions of metastatic serous OC. Drug effects in organoids proved more diverse and rather refractory [111]. Patient-derived HGSC organoids were developed with a high success rate and used for functional profiling of DNA repair, accurately predicting clinical response of patients to DNA repair inhibitors [112]. More recently, comprehensive establishment of 56 OC organoid lines from 32 OC cases was reported [113]. Although two-thirds of organoids were derived from serous OC in this study, it achieved a success rate of 65%. Notably, it covered all the four major subtypes for the first time and basic features of the original tumors were mostly retained. Moreover, organoids established from normal fallopian tube and OSE were subjected to p53 inactivation to model HGSC. We also established an efficient organoid culture method for ovarian and endometrial tumors [114]. We modified a Matrigel bilayer organoid culture protocol (MBOC), originally developed for a murine carcinogenesis model ex vivo [115], to cope with the digestion-resistant nature of OC. A total of nine OC-organoid lines were established from not only HGSC, but also mucinous, endometrioid carcinoma, and even borderline or early-stage tumors. Propagated organoids retained many aspects of the original tumors, including histopathological features, mutation profiles, and intra-tumoral heterogeneity. Drug response assay was also feasible using organoid-derived spheroids. Thus, the organoid platform might be potentially promising in drug discovery and personalized medicine. 

To better understand the biological features of OC, thorough elucidation of interactions between tumor cells and the microenvironment is a critical issue. However, standard organoid culture consists of only epithelial cells and lacks such interactions. To address this issue, 3D organotypic models that reproduce a similar situation to that in vivo have been developed [116]. Specifically, the microenvironment of OC was first reconstituted in vitro, by using omentum-derived primary mesothelial cells and fibroblast at early passages. OC cells were then plated to examine the mechanisms underlying attachment and invasion of OC. Another model is a 3D microfluid-based model that dynamically reconstitutes interactions of OC with mesothelial cells during peritoneal dissemination. In this platform, living cells are infused into micrometer-sized chambers, enabling accurate control of the cellular microenvironment [117]. While these organotypic co-culture models are complex systems and may not be ideal for robust propagation of organoids, their physiological features might be suitable for drug discovery in the next-generation. 

## 5. Conclusions

Until very recently, use of patient-derived OC as preclinical models has been quite limited because the success rate of primary cultures was low, and even if cell lines are established, features often differ from those of the original tumors. Recent remarkable advances in 3D culture technique have allowed us to reconstitute many features of the original tumors in an in vitro setting. In the light of rapid implementation of precision medicine, which largely depends on genomic information of each cancer, establishment of patient-derived cell-based assays will be of critical relevance. Among various platforms, the organoid might be the most powerful tool in high-throughput drug screening and establishment of xenografts. Now that OC organoids are becoming available, its nation-wide banking will be readily started as a valuable resource for OC researches. Collectively, OC organoids per se and organoid-derived PDX will likely contribute to the development of novel therapies as well as elucidation of its pathogenesis.

## Figures and Tables

**Table 1:** List of studies on primary organoid culture of ovarian cancers.

Reference	Success		Histological Type	Patient’s Material	PDX
	Case (*n*)	Rate (%)			
[102]	1	100	N.D.	N.D.	N.T.
[111]	9	N.D.	SC	Tissue, Ascites, Pleural effusion	Yes
[112]	23	80–90	HGSC, CS	Tissue, Pleural effusion	N.T.
[113]	32	65	MBT, SBT, CCC, EMC, MC, LGSC, HGSC	Tissue, Ascites, Pleural effusion	Yes
[114]	9 (4*, 5^#^)	60 (44*, 83^#^)	BBT, SBT, EMC, MC, HGSC	Tissue	Yes

SC, serous carcinoma; HGSC, high-grade SC; LGSC, low-grade SC: CS, carcinosarcoma, CCC, clear cell carcinoma, EMC; endometrioid carcinoma; MBT, mucinous borderline tumor, SBT, serous borderline tumor; BBT, borderline Brenner tumor; N.D., not described; N.T., not tested.; PDX, patient-derived xenograft; *standard Matrigel bilayer organoid culture (MBOC); #modified MBOC.
